# Identification of lncRNAs and Their Regulatory Relationships with mRNAs in Response to *Cryptococcus neoformans* Infection of THP-1 Cells

**DOI:** 10.1155/2022/5532118

**Published:** 2022-03-26

**Authors:** Rui Gao, Guotai Yao, Xiaolie Wang, Yilin Wang, Wenting Lin, Liang Teng, Yan Wang, Yi Jin, Zhongzhi Wang, Jianghan Chen

**Affiliations:** ^1^Department of Dermatology, Changzheng Hospital, Naval Medical University, No. 415 Fengyang Road, Shanghai 200003, China; ^2^Department of Dermatology, General Hospital of Southern Theatre Command of PLA, Guangzhou 510010, China; ^3^Department of Dermatology, Shanghai Skin Disease Hospital, Tongji University School of Medicine, Shanghai 200443, China; ^4^Department of Dermatology, Shanghai Fourth People's Hospital, Tongji University School of Medicine, Shanghai 200434, China

## Abstract

**Aims:**

Cryptococcosis is an invasive fungal disease that is associated with an increasing prevalence along with a very high fatality and is primarily caused by Cryptococcus. However, its mechanism to cause pathogenicity is not yet completely understood. In this study, we aim to screen the lncRNA markers in human monocytic (THP-1) cells infected by *Cryptococcus neoformans* (*C. neoformans*) through high-throughput sequencing technology and to explore its effects on biological functions.

**Methods:**

We initially conducted an lncRNA microarray analysis of the THP-1 cells infected by C. neoformans and normal THP-1 cells. Based upon these data, RT-qPCR was used to verify the expressions of the selected lncRNAs and mRNAs. We then performed functional and pathway enrichment analyses. Lastly, target prediction was performed by using the lncRNA target tool which was based on the differentially expressed lncRNAs.

**Results:**

We determined 81 upregulated and 96 downregulated lncRNAs using microarray. In addition, the profiling data showed 42 upregulated and 57 downregulated genes and discovered that neuroactive ligand-receptor interaction, tyrosine metabolism, and phenylalanine metabolism are extremely impaired in the regulation of *C. neoformans* infection. GO enrichment analysis of the 99 differentially expressed mRNAs exhibited that these modules showed different signaling pathways and biological mechanisms like protein binding and metal ion binding. Moreover, lncRNAs and mRNAs were analyzed for their coexpression relations. A qRT-PCR analysis confirmed that the expression of the top 10 differently expressed mRNA and lincRNA. The expressions of the lncRNAs after *C. neoformans* infection in THP-1 cells were detected by RNA-sequence, suggesting that microarray analysis could reveal lncRNAs having functional significance that might be linked with the progression of patients.

**Conclusion:**

The current study analyzed the differential lncRNAs and mRNAs in *C. neoformans* infection and predicted the corresponding pathways and their correlations that can offer new potential insights into the mechanistic basis of this condition.

## 1. Introduction

Epidemiological studies have shown that cryptococcosis is a common cause of dangerous and life-threatening invasive fungal disease. The occurrence of cryptococcal disease is exceptionally high in developing countries, where it is responsible for almost a third of all deaths in patients with AIDS/HIV, exceeding the death rates of tuberculosis in some regions. With the establishment of immunosuppressive therapy for malignant tumors and autoimmune diseases, the high-risk population for cryptococcosis is expanding [1]. In immunocompromised patients, *Cryptococcus* spreads from the lungs to the brain, thereby leading to fatal cryptococcal meningitis, which is difficult and costly to treat. The largest epidemiological evaluation of cryptococcal meningitis (CM) in the US unveiled that the total occurrence of cryptococcosis is diminishing, but the epidemiology is complex and changing, and CM is still a significant danger [2]. Therefore, it is immensely important to continue research on the development of better treatments.

The monocyte-macrophage lineage is a group of important inflammatory cells in the first line of the body's defense system, and it broadly protects against invading fungi and other microorganisms. Macrophages are its chief constituents; they have variable functions mostly responsible for host defense and provide immunity against invading microorganisms such as viruses, bacteria, parasites, and fungi [3]. THP-1 is a mononuclear cell line in human leukemia and is widely used in studies on the functions, mechanisms, signaling pathways, nutrition, and drug transport of mononuclear/macrophage cells. This cell line has become a common model to estimate modulation of monocyte and macrophage activities. Considering the importance of monocytes in the immune response, further examination of immune-based mechanism in monocytes, especially when they are exposed to *Cryptococcus neoformans*, is relevant.

Our previous studies found that after the infection of THP-1 cells by *C. neoformans*, the expression of a variety of miRNAs changed. Specifically, the expression of miR-146a was upregulated, which negatively regulated the NF KB pathway, thereby playing a role in the inflammatory response [4]. Moreover, miRNA-30c-5p is known to suppress inflammatory, apoptotic, and autophagic response via modulation of the eIF2*α*/ATF4 network [5]. It has been found the differential long noncoding RNA (lncRNA) profile in cryptococcal meningitis patients and DPY19L1p1 can be employed for prognostic assessment and disease diagnosis [6]. The importance of lncRNAs as transcriptional noise remained neglected for the last 10 years, but it has now been recognized [7]. The disease processes could be revealed by the crosstalk of coding and noncoding RNAs, which may provide opportunities for novel therapeutics [8]. Furthermore, lncRNAs regulate the profile of gene expression and act as capital transcriptional regulators that affect their extracellular environments [9]. Similarly, lncRNAs are currently believed to be the chief regulatory molecules for the expression of transcripts in both humoral and cellular immune cells against invading microbes [10]. Nevertheless, current evaluations of the lncRNA mechanism in cryptococcal infections are not complete. Therefore, an ongoing examination of lncRNAs may produce novel findings and provide insights for better therapies of cryptococcal infections or CM. Most patients with CM do not receive prompt diagnosis and effective therapy owing to the absence of apparent symptoms in the early stage of disease. Unfortunately, the molecular pathogenic mechanisms are not completely clear. Microarray analysis is commonly employed to identify differentially regulated genes in patients suffering from different diseases. This analysis can also uncover gene expression, interactions, linkage, and aid in mapping [11, 12]. This technology is also beneficial in understanding gene association and mapping. However, there have been no studies on the *C. neoformans*-infected THP-1 cells' transcriptome versus normal controls. Hence, our goal here was to determine differentially expressed gene (DEG), lncRNA, and mRNA networks and aberrant networks that potentially regulates progression of THP-1 cell infection. Our conclusions will facilitate novel understanding of the functional modulatory networks of cryptococcal infections and may show promising clues to control infection progression.

## 2. Materials and Methods

### 2.1. Cell Culture

We cultured THP-1 cell line (Stem Cell Bank/Stem Cell Core Facility, Shanghai Institute of Biochemistry and Cell Biology, Chinese Academy of Sciences) in a medium of RPMI 1640 (Gibco Company, USA) complemented with 100 units/ml penicillin in addition to 10% fetal bovine serum along with 100 *μ*g/ml streptomycin (P/S) while employing an incubator with 5% CO_2_ at a temperature of 37°C. The media was removed and replenished every 2 days, and when 80–90% confluency was achieved, cells were typically subcultured. Moreover, six-well plates were employed for seeding THP-1 cells at 4 × 10^6^/well in growth medium and kept in an incubator set at a temperature of 37°C along with 5% CO_2_.

### 2.2. Cryptococcal Intervention

The THP-1 cells cultured with 2 ml 10% FBS high-glucose RPMI1640 complete medium without 1% penicillin/streptomycin were converted to macrophages with PMA (100 ng/ml, 48 h), and then, they were incubated together with a strain of *C. neoformans* (WM148) that was heat-killed for 0 and 6 h. In all experiments, the cells were exposed to WM148 at an MOI of 1 : 5.

### 2.3. RNA Isolation and Sequencing

When 85% confluency was reached (it contained almost 10^7^ cells), the cells were stimulated with 5 × 107 inactivated *C. neoformans* (WM148). The ratio of WM148 and cells was 5 : 1. The cells were assigned to 0 h and 6 h groups representing the control and intervention groups, respectively [4]. The supernatants and cells were collected at 0 h and 6 h following WM148 infection (*n* = 3 per group). Six samples were required, three of them were the intervention groups and the other half were control groups. Furthermore, TRIzol (Invitrogen, CA, USA) was utilized for extracting the overall RNA from each of the samples by complying with the company's instructions. Similarly, Bioanalyzer 2100 in conjunction with RNA 6000 Nano LabChip Kit (Agilent, CA, USA) that possessed RIN value greater than 7.0 was employed for the analysis of total RNA purity in addition to its quantification. Moreover, for depleting ribosomal RNA, we utilized almost 5 *μ*g of the entire RNA as described in the Epicentre Ribo-Zero Gold Kit (Illumina, San Diego, USA) directions. In addition, purification was followed by the fragmentation of poly(A)- or poly(A)+RNA into small fragments with the help of divalent cations using raised temperature. Similarly, for the construction of the final cDNA library, we performed reverse transcription of the chopped RNA fragments while complying with the instructions in the mRNA-Seq sample preparation kit (Illumina, San Diego, USA). For the paired-end libraries, the average size of the insert was 300 bp (±50 bp). Furthermore, paired-end sequencing was accomplished through Illumina Hiseq 4000 (lc-bio, China) according to the protocol suggested by the vendor. The kits were from Shanghai Biotechnology Corporation.

### 2.4. lncRNA Identification

In the first step, we discarded the transcripts that were found to be overlapping with known mRNAs and also the ones whose length was less than 200 bp. In the next step, the transcripts having coding ability were predicted by utilizing CPC [13] and CNCI [14]. After removing the transcripts that possessed a CPC score < −1 in addition to CNCI score < 0, lncRNAs were obtained.

### 2.5. Target Gene Prediction and GENE Function Enrichment Analysis

Gene Ontology (GO) function enrichment analysis of DEGs and differentially regulated IRGs was performed via the R package “clusterProfiler” [15]. This included biological process (BP), molecular function (MF), and cellular components (CC). We also conducted pathway analysis using Kyoto Encyclopedia of Genes and Genomes pathways (KEGG). The Benjamini and Hochberg formula was used to adjust the *P* value, and <0.05 was set as the significance threshold. We had to predict lncRNA cis-target genes of in order to investigate the functions associated with lncRNAs, as lncRNAs may have a cis function on adjacent downstream genes. Therefore, here, Perl script was used to select 100,000 bp upstream as well as downstream coding genes. After that, target genes for lncRNAs were functionally analyzed employing the in-house scripts. Significance has been exhibited as a *P* value ≤ 0.05.

### 2.6. Confirmation via Quantitative Real-Time PCR (qRT-PCR)

We isolated total RNA from the heart tissue, prior to conversion to cDNA. Subsequently, lncRNAs were assessed via qRT-PCR with the help of Ribo qPCR SYBR Green Master Mix (Ribo Biotech, Guangzhou, China) and a 7900HT Fast Real-Time PCR system (Life Technologies, Carlsbad, CA, USA). The qRT-PCR parameters were as follows: 95°C for 10 min, 40 cycles of 95°C for 5 s, 60°C for 30 s, and 72°C for 30 s. We next designed the lncRNA primers and Ribo (Guangzhou, China) conducted primer synthesis (Supplemental Table S1). The qRT-CR reaction was done in a 20 *μ*l solution that contained 2 *μ*l 100 ng/ml sample cDNA, 10 *μ*l 2x SYBR Green Master Mix, 0.8 *μ*l each of 5 *μ*M forward and reverse primers, and 6.4 *μ*l of RNase/DNase-free water. The relative gene levels were computed with the 2^-*ΔΔ*Ct^ formula [16], and GAPDH was employed as an internal control.

### 2.7. Data Preprocessing and DEG Identification

Using the robust multiarray technology, we corrected the preliminary data against background, quantile normalization, and log transition [17]. During early processing, Entrez's Gene ID converter was used to alter the specific gene symbols in the probe IDs [18]. In cases when the same gene contributed in multiple samples, the average value was computed and regarded as the final expression value. For the analysis of the original expression data, we employed the freely available tool GEO2R, which includes both R/Bioconductor and Limma package v3.26.8 [19, 20]. The GEO2R inbuilt assessments like *t*-test and Benjamini and Hochberg (false discovery rate) were employed for the calculation of *P* value and FDR to identify DEGs among FH patients and controls [21]. To identify DEGs from the dataset, we adjusted the criteria as follows: ∣ log (fold change)  | >1 and *P* < 0.05. Highly expressed DEGs were identified with logFC ≥ 1 and scarcely expressed DEGs with logFC ≤ −1 (see supplementary file). The RStudio (v1.2.5019) and library Calibrate package were utilized for the generation of a volcano plot. The corresponding DEGs were obtained from a dataset, for further analysis. We constructed a heat map with a heat mapper webserver 2 to express the relative gene levels. The inbuilt average linkage clustering was employed for the calculation of hierarchical clustering, and the Euclidean algorithm was employed for the determination of distance between rows and columns [22].

### 2.8. Statistical Analysis

Data expressed as mean ± standard deviation (SD). A *t*-test was employed for intergroup data comparisons. *P* value < 0.05 was set as the significance threshold. All data analyses was conducted in the GraphPad Prism software (La Jolla, CA, USA).

## 3. Results

### 3.1. Overview of RNA Sequencing

Six libraries of cDNA were constructed (Case1, Case3, Case5, Control2, Control4, and Control6). Annotation of 58,825 genes along with 208,460 transcripts was carried out. Each sample contained raw data that were found to be higher than 100,000,000; 109,588,370 clean reads on average in each sample were attained after carrying out quality control. Each of the sequencing libraries yielded clean data that accounted for approximately 96% of the raw data, and Q20 and Q30 reads accounted for more than 93%. Out of all the clean reads, almost 97% were mapped to the human reference genome (Table 1), indicating that the data obtained by sequencing are good and, therefore, can be further used for data analysis.

### 3.2. The Comparison of Transcript Characteristics

**Figure 1:**
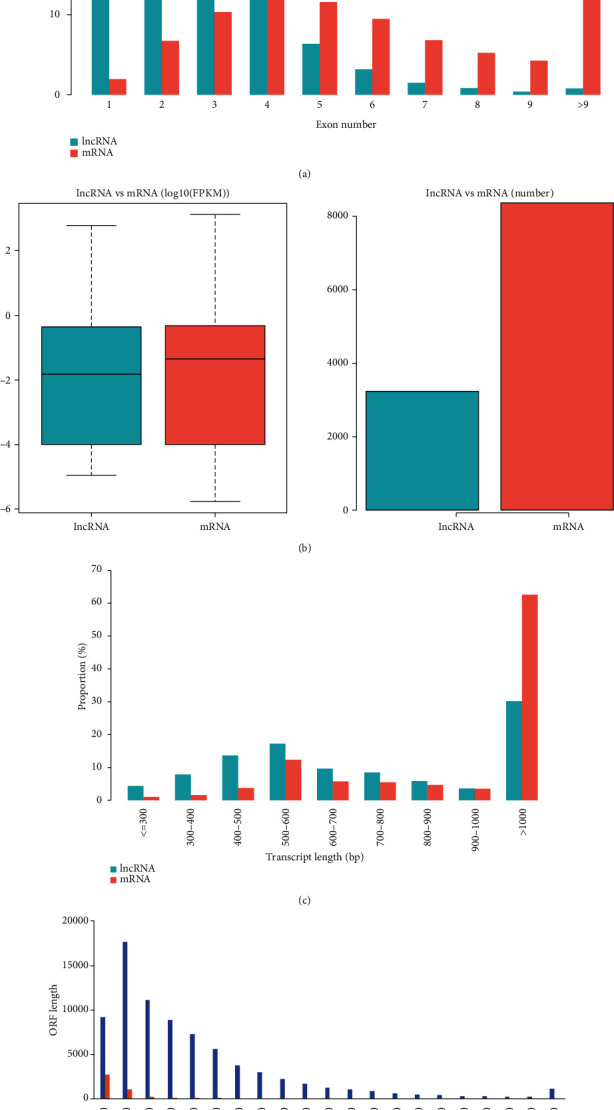
The characteristic comparison of transcripts. (a) The distribution for exon number of lncRNAs along with mRNAs is given. (b) The expression level in addition to several lncRNAs besides mRNAs. (c) The distribution of varying length lncRNAs along with mRNAs has been given. (d) The distribution of lncRNAs along with mRNAs with varying length ORF. Moreover, mRNAs always carried longer ORF than lncRNAs. mRNA: messenger RNA; lncRNA: long noncoding RNA.

Novel lncRNAs were identified in the current study with the help of CNCI that includes GitHub (GitHub, CA, Version 2, San Francisco, USA) along with CPC software (CBI, Version 2, USA). The exon number for these lncRNAs was 3.5 on average, which was much smaller than 10.6 exons on average for mRNAs (Figure 1(a)).  Figure 1(b) shows the level of expression and several lncRNAs and mRNAs. In the following figures, comparisons of full length along with open-reading frame length distribution of mRNAs and lncRNAs are shown. We found that mRNAs were more conserved in sequences than lncRNAs (Figures 1(c) and 1(d)).

### 3.3. Differential Expression Profiles and Verification of lncRNAs and mRNAs between C. neoformans (WM148)-Infected THP-1 Cells and Controls

**Figure 2:**
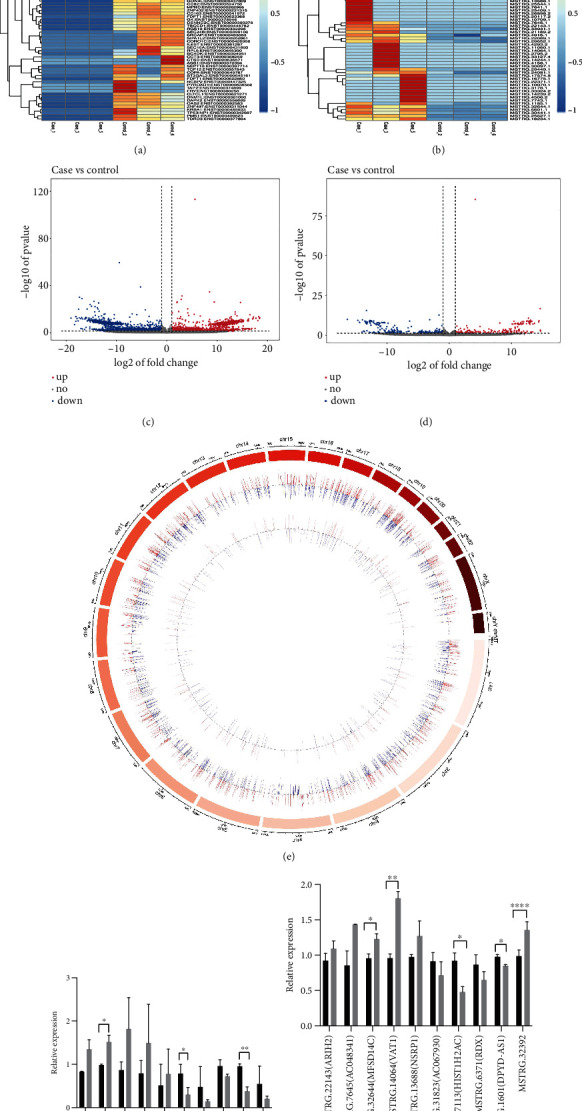
lncRNAs along with mRNAs with their differential expression between THP-1 cells (*n* = 3) infected with *Cryptococcus neoformans* and uninfected control groups (*n* = 3). (a and b) Heat map of mRNA along with novel lncRNA expression. (c and d) Volcano plot of mRNA besides novel lncRNA expression; red dots depict upregulation of RNAs with *P* value < 0.05 along with a fold change ≥ 1, while blue dots illustrate downregulation of RNAs with *P* value < 0.05 along with a fold change ≤ −1. (e) Distribution of chromosomes for lncRNAs along with mRNAs with their differential expression are demonstrated, respectively, by the outer circle to the inner circle. Moreover, downregulation is represented by green, upregulation is represented by red, and gene enrichment is represented by the height of the bars. Case: *C. neoformans*-infected THP-1 cells; Control: normal THP-1 cells. (f and g) The top 10 differentially expressed mRNAs and lncRNAs for the real-time PCR validation of relative expression in *C. neoformans* (WM148)-infected THP-1 cells compared to controls. 0 h represents THP-1 cells without treated with WM148; 6 h represents THP-1 cells were treated with WM148 for 6 h. All results are expressed as mean ± SD from three independent experiments. ^∗^*P* < 0.05 and ^∗∗^*P* < 0.01 compared with the control group (0 h).

In the present study, 4151 mRNAs (out of which 2007 were upregulated and 2144 were downregulated) along with 531 novel lncRNAs (having 244 upregulated lncRNAs and 287 downregulated lncRNAs) showed differential expression (datasets are available on request). Those mRNAs and lncRNAs showing a change in expression of at least 2.0-fold and *P* < 0.05 were recognized to exhibit a significantly differential expression. In total, 99 mRNAs (42 upregulated and 57 downregulated) (Figures 2(a) and 2(c)) along with 177 lncRNAs (81 upregulated and 96 downregulated) (Figures 2(b) and 2(d)) were determined. The top 10 remarkably expressed mRNAs and lncRNAs in the *C. neoformans*-infected group are shown in Tables 2 and 3, respectively. Moreover, we generated a circos plot to reveal the chromosomal allocation of the mRNAs and lncRNAs exhibiting differential expression (Figure 2(e)). The top 10 differentially expressed mRNAs and lncRNAs were selected for the real-time PCR validation of the relative expression in *C. neoformans* (WM148)-infected THP-1 cells. The mRNAs (Figure 2(f)) for small integral membrane protein 34B (SMIM34B), insulin-like growth factor II (IGF2), hypermethylated in cancer 1 (HIC1), transient receptor potential cation channel subfamily V member 3 (TRPV3), and Rhox homeobox family member 2 (RHOXF2) were highly expressed, and IGF1 was significantly highly expressed. The mRNAs (Figure 2(f)) for olfactory receptor family 4 subfamily C member 6 (OR4C6), prostate and testis expressed 2 (PATE2), C-C motif chemokine 18 (CCL18), epithelial cell adhesion molecule (EPCAM), and glial cells missing homolog 2 (GCM2) were poorly expressed, among which OR4C6 and EPCAM were significantly highly expressed. These results were consistent with the microarray analysis results. The lncRNAs (Figure 2(g)) for MSTRG.22143 (ARIH2), MSTRG.7645 (AC048341), MSTRG.32644 (MFSD14C), MSTRG.14064 (VAT1), and MSTRG.13688 (NSRP1) were found to be highly expressed, among which MSTRG.32644 (MFSD14C) and MSTRG.14064 (VAT1) were significantly highly expressed. The lncRNAs (Figure 2(g)) for MSTRG.31823 (AC067930), MSTRG.27113 (HIST1H2AC), MSTRG.6371 (RDX), and MSTRG.1601 (DPYD-AS1) were poorly expressed, among which MSTRG.27113 (HIST1H2AC) and MSTRG.1601 (DPYD-AS1) were significantly poorly expressed. These results were consistent with the microarray analysis results. However, MSTRG.32392 was found to be highly expressed, which was contrary to the microarray analysis results.

### 3.4. GO and KEGG Pathway Analyses of mRNAs

**Figure 3:**
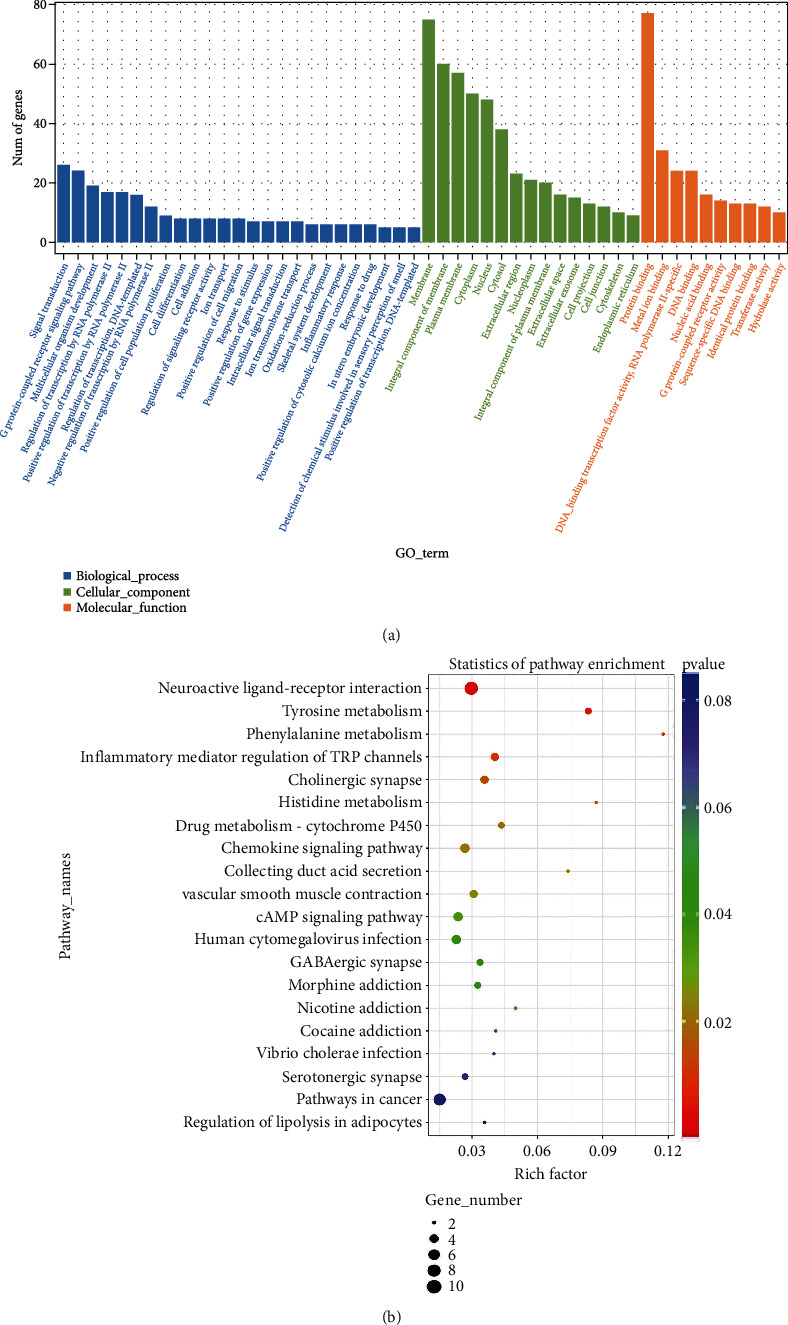
Analyses of GO along with KEGG for the differential mRNA expression profile. (a) GO and (b) KEGG.

In order to investigate the possible biological properties of the mRNAs showing differential expression in *C. neoformans*-infected THP-1 cells, we conducted GO and KEGG pathway enrichment analyses. Figures 3(a) and 3(b) show the molecular functions, cellular components, and biological processes linked with these mRNAs. The five primary GO terms involved include protein binding, metal ion binding, DNA-binding transcription factor activity, DNA binding, and nucleic acid binding. The five primary KEGG pathways involved include neuroactive ligand-receptor interaction, tyrosine metabolism, phenylalanine metabolism, inflammatory mediator regulation of TRP channels, and cholinergic synapse.

### 3.5. Coexpression of lncRNAs and mRNAs

**Figure 4:**
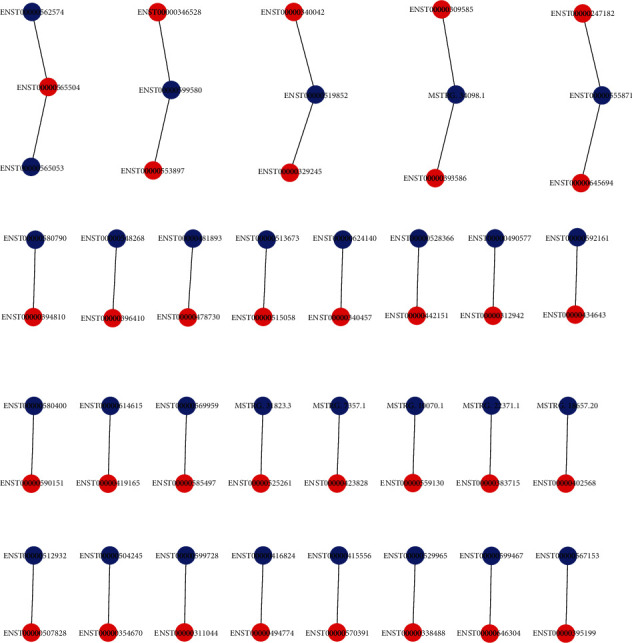
Cis-regulation network of mRNA and lncRNA with Pearson′s correlation > 0.8 or < −0.8. Red dot represents mRNA, and blue dot represents lncRNA.

The location relationship was used to predict the genes targeted for cis-regulation by lncRNAs. In order to identify the candidate lncRNAs, screening of 100 kb upstream sequences along with a screening of 100 kb downstream sequences was carried out. As already illustrated in a previous study, the position frequency matrix leads to the development of connectivity or enrichment. The results included only those genes that exhibited tight correlation expression profiles (Pearson's targets *r* ≥ 0.8). Target prediction was performed by using the lncRNA target tool which was based on the differentially expressed lncRNAs. Eventually, we identified 34 pairs of lncRNA-mRNA in the case group compared with the control group (Figure 4).

## 4. Discussion

Cryptococcosis is triggered by *C. neoformans* or *C. gattii*. *C. neoformans* infection is a substantial pervasive fungal infection that induces significant morbidity and mortality [23]. Macrophages participate in the host defense and immunity against microorganisms such as fungi and other microorganisms, and they are a pivotal part of the immune system with multiple functions. In recent times, epigenetics garnered much attention for its contribution to a wide variety of cellular processes.

Noncoding RNAs include a multitude of small or long RNAs with district characteristics and roles. Small noncoding RNAs include microRNAs, small nucleolar RNAs (snoRNAs), and small nuclear RNAs (snRNAs). Long noncoding RNAs (lncRNAs) include RNA molecules with over 200 nucleotides, often reaching up to 100 kb [24].

lncRNAs are present in both nuclear and cytosolic fractions and are involved in the etiology of numerous human diseases, especially cancers [25]. Furthermore, it has recently been discovered that lncRNAs contribute to the modulation of the immune system, in addition to gene regulation and a variety of important biological processes [10, 26, 27]. Nevertheless, limited information is currently available regarding the significance of lncRNAs in *C. neoformans* infection.

Currently, there is only one report of elevated lncRNA DPY19L1p1 expression in patients with CM [6]. However, there is not enough evidence to show what role lncRNAs play in *C. neoformans* infection. This study was aimed at identifying and characterizing lncRNAs in *C. neoformans* and at deciphering the mechanisms regulating their expression.

Here, we employed RNA sequencing to examine the expression of lncRNAs in THP-1 cells infected by *C. neoformans* and normal THP-1 cells to scrutinize the function of lncRNAs in *C. neoformans* infection. As far as we are aware, this is the first reported investigation on lncRNA expression profiling of *C. neoformans* infection in THP-1 cells. We found 81 upregulated and 96 downregulated lncRNAs in addition to 42 upregulated and 57 downregulated mRNAs in the infected cells, relative to controls (a *P* value of < 0.05). Subsequently, we utilized bioinformatics methods, such as GO term enrichment and cellular network enrichment, to systematically investigate the identified DEGs. We implemented GO and KEGG analyses to determine MF, CC, BP, and pathways involving DEGs via the online DAVID technology. The chief pathways controlled by these differential mRNAs include tyrosine metabolism, neuroactive ligand-receptor binding, phenylalanine metabolism, cholinergic synapse, and inflammatory mediator modulation of TRP channels. Most of the differentially regulated lncRNAs in patients with CM were intronic lncRNAs. lncRNAs having related functions can interact with each other or they can also have alike network data profiles [28, 29].

*C. neoformans* activates the EPH-tyrosine kinase network via a CD44-dependent EphA2 phosphorylation. This promotes cluster formation and internalization of EphA2 receptors [30]. Protein tyrosine phosphatases (PTPs) are stress-responsive genes that are regulated by MAPK Hog1 and transcription factor Atf1. Ptp2 prevents excessive Hog1 phosphorylation and mediates vegetative growth, sexual differentiation, stress responses, antifungal drug resistance, and virulence factor modulation via a negative-feedback loop involving the HOG network [31]. Pear fruits (*Pyrus pyrifolia* L. cv. Yali) exposed to various elicitors like salicylic acid, oxalic acid, calcium chloride, and antagonistic yeast *Cryptococcus laurentii* were shown to regulate defense responses. The data revealed that elicitors markedly enhanced enzymatic activity of phenylalanine ammonia lyase [32]. TRP channels are responsive to a wide range of harmful physical and chemical stimuli, which, in turn, augment intracellular cation concentrations. The TRP channel has multifold regulation in immune cells, ranging from cell migration and phagocytosis modulation to the synthesis and release of inflammatory factors. Moreover, these channels also promote a robust crosstalk between epithelial cells, neuronal tissue, and immune cells that work together to regulate immune responses to damaged tissue or infection [33].

We verified the top 10 differently expressed mRNAs and lncRNAs by qPCR, and based on our analysis, the qPCR data were approximately consistent with the microarray analysis data. It has been found that IGF2 in low dose acts through IGF2R and alleviates colitis via promoting anti-inflammatory macrophages, suggesting that IGF2 family strongly controls generation of anti-inflammatory macrophages and may provide novel strategies for the treatment of inflammatory diseases [34]. These evidence indicate that IGF2 is critical for the pathogenesis of cryptococcal meningitis, but further research is needed. We used transcriptome sequencing and bioinformatics analysis to identify DEGs during the infection of THP-1 cells by *C. neoformans*, so as to analyze the relevant roles of these genes in the development of CM and determine whether they can be used as potential molecular markers. Network analysis of transcriptomics data for the prediction and prioritization of inflammatory-associated biomarkers for CM by bioinformatics approach [35]. A coexpression system between mRNAs and lncRNAs was created to anticipate the possible functions of lncRNAs. Overall 34 coexpression relationships were shown between mRNAs and lncRNAs, implying that many lncRNAs may have significant roles in the progression of *C. neoformans* infection and immune regulation. Thus, the outcome of our study imparts a wider comprehension of the interaction between the immune system and *Cryptococcus*. The majority of lncRNAs and expression changes reported here have been poorly studied to date. Hence, the exact mechanism involved with these lncRNAs requires more research.

## 5. Conclusion

Our study through RNA-Seq analysis showed, for the very first time, that lncRNAs had differential expression among *C. neoformans* infection in THP-1 cells and normal THP-1 cells. A total of 177 lncRNA transcripts showed differential expression between the infected group and control group: 81 lncRNAs were found to be upregulated, and 96 were found to be downregulated. In order to investigate the function of lncRNAs, an integrative approach was applied. lncRNAs showing differential expression might be involved in the regulation of *C. neoformans* infection. We verified the top 10 differently expressed mRNAs and lncRNAs by qPCR and found that the qPCR results were approximately consistent with the microarray analysis results except lncRNA MSTRG.32392. However, there are still some limitations in this study; we only perform cis-regulation predictions on the upstream and downstream 100 k mRNA and lncRNA. The experimental verification of the functions of these lncRNAs and mRNAs is required to be carried out in the future via knockout or overexpression methods. Therefore, the results of this study not only yield an extensive analysis of lncRNAs but also set up a strong foundation to study the mechanisms and functions of lncRNAs in *C. neoformans* infection.

## Figures and Tables

**Table 1:** Quality statistics of different sample sequencing data.

Sample	Raw data		Valid data		Valid ratio (%)	Q20 (%)	Q30 (%)	Mapped ratio (%)
	Read	Base	Read	Base				
Case 1	100907820	10.09G	97337470	9.73G	96.46	99.65	93.62	96.75%
Case 3	100907820	10.14G	97777644	9.78G	96.47	99.77	94.53	97.22%
Case 5	111127214	11.11G	107057624	10.71G	96.34	99.73	94.29	97.03%
Control 2	127793504	12.78G	123059382	12.31G	96.3	99.72	93.95	97.00%
Control 4	129181238	12.92G	124703732	12.47G	96.53	99.79	95.11	97.42%
Control 6	111883148	11.19G	107594368	10.76G	96.17	99.57	93.04	96.47%

Note: Case represents *C. neoformans*-infected THP-1 cells; control represents *C. neoformans*-uninfected THP-1 cells.

**Table 2:** The 10 upregulated and downregulated mRNAs in *C. neoformans*-infected THP-1 cells.

Upregulated mRNAs			Downregulated mRNAs		
Transcript ID	*P* value	Log^2^ fold change (case vs. control)	Transcript ID	*P* value	Log^2^ fold change (case vs. control)
ENSG00000278961 (SMIM34B)	1.00 × 10^−7^	9.83	ENSG00000181903 (OR4C6)	1.40 × 10^−3^	-9.3
ENSG00000167244 (IGF2)	2.33 × 10^−3^	7.26	ENSG00000196844 (PATE2)	2.87 × 10^−4^	-9.17
MSTRG.13124 (HIC1)	4.69 × 10^−3^	4.32	ENSG00000275385 (CCL18)	6.71 × 10^−4^	-9.16
ENSG00000167723 (TRPV3)	1.70 × 10^−3^	4.09	ENSG00000119888 (EPCAM)	2.80 × 10^−3^	-8.93
ENSG00000131721 (RHOXF2)	3.57 × 10^−3^	3.49	MSTRG.26977 (GCM2)	1.05 × 10^−3^	-8.32
MSTRG.11170 (NRG4)	1.28 × 10^−7^	2.92	ENSG00000069535 (MAOB)	6.80 × 10^−3^	-8.31
MSTRG.16004 (TIMM29)	1.29 × 10^−16^	2.7	ENSG00000102239 (BRS3)	2.58 × 10^−3^	-8.18
MSTRG.295 (KAZN)	1.22 × 10^−4^	2.33	ENSG00000174460 (ZCCHC12)	3.15 × 10^−3^	-8.06
MSTRG.10244 (CHRM5)	1.26 × 10^−4^	2.28	MSTRG.5103 (OR51M1)	7.56 × 10^−4^	-7.76
MSTRG.17477 (ATP6V1C2)	2.64 × 10^−14^	1.99	ENSG00000187486 (KCNJ11)	4.45 × 10^−3^	-7.54

Note: Case represents *C. neoformans*-infected THP-1 cells; control represents *C. neoformans*-uninfected THP-1 cells.

**Table 3:** The 10 upregulated and downregulated novel lncRNAs in *C. neoformans*-infected THP-1 cells.

Upregulated lncRNAs			Downregulated lncRNAs		
Transcript ID	*P* value	Log^2^ fold change (case vs. control)	Transcript ID	*P* value	Log^2^ fold change (case vs. control)
MSTRG.22143 (ARIH2)	1.98 × 10^−3^	14.69	MSTRG.31823 (AC067930)	1.18 × 10^−10^	-16.37
MSTRG.7645 (AC048341)	5.74 × 10^−4^	14.66	MSTRG.27113 (HIST1H2AC)	4.58 × 10^−10^	-14.68
MSTRG.32644 (MFSD14C)	1.87 × 10^−17^	14.64	MSTRG.6371 (RDX)	2.366 × 10^−10^	-13.94
MSTRG.14064 (VAT1)	2.90 × 10^−8^	13.71	MSTRG.1601 (DPYD-AS1)	3.12 × 10^−3^	-13.66
MSTRG.13688 (NSRP1)	7.49 × 10^−8^	13.48	MSTRG.32392	3.78 × 10^−3^	-13.65
MSTRG.10709 (CCPG1)	1.71 × 10^−9^	13.31	MSTRG.27544 (BTBD9)	1.49 × 10^−9^	-13.55
MSTRG.4158 (HERC4)	3.79 × 10^−10^	13.08	MSTRG.29230 (PSPHP1)	7.93 × 10^−9^	-13.42
MSTRG.25409 (GPBP1)	6.13 × 10^−9^	13.04	MSTRG.7357 (TMBIM6)	2.31 × 10^−8^	-13.37
MSTRG.11060 (PKM)	9.82 × 10^−8^	12.99	MSTRG.26157 (AC010240)	2.26 × 10^−9^	-13.36
MSTRG.30097 (EXOC4)	9.56 × 10^−10^	12.96	MSTRG.11028 (PIAS1)	2.92 × 10^−16^	-13.3

Note: Case represents *C. neoformans*-infected THP-1 cells; control represents *C. neoformans*-uninfected THP-1 cells.

## Data Availability

The data used to support the findings of this study are available from the corresponding author upon request.

## Supplementary Materials

Supplementary Materials Supplementary 1. Identified lncRNAs and mRNAs that are differentially expressed along with the logFC values and *P* values.

## References

[1] Szymczak W. A., Davis M. J., Lundy S. K., Dufaud C., Olszewski M., Pirofski L. A. (2013). X-Linked immunodeficient mice exhibit enhanced susceptibility to Cryptococcus neoformans infection. *MBio*.

[2] Pyrgos V., Seitz A. E., Steiner C. A., Prevots D. R., Williamson P. R. (2013). Epidemiology of Cryptococcal meningitis in the US: 1997–2009. *PLoS One*.

[3] Murray P. J., Wynn T. A. (2011). Protective and pathogenic functions of macrophage subsets. *Nature Reviews Immunology*.

[4] Chen H., Jin Y., Chen H., Liao N., Wang Y., Chen J. (2017). MicroRNA-mediated inflammatory responses induced by Cryptococcus neoformans are dependent on the NF-*κ*B pathway in human monocytes. *International Journal of Molecular Medicine*.

[5] Jin Y., Yao G., Wang Y. (2020). MiR-30c-5p mediates inflammatory responses and promotes microglia survival by targeting eIF2*α* during *Cryptococcus neoformans* infection. *Microbial Pathogenesis*.

[6] Zhang L., Fang W. J., Zhang K. (2019). Long noncoding RNA expression profile from cryptococcal meningitis patients identifies DPY19L1p1 as a new disease marker. *CNS Neuroscience & Therapeutics*.

[7] Jandura A., Krause H. M. (2017). The new RNA world: growing evidence for long noncoding RNA functionality. *Trends in Genetics*.

[8] Salmena L., Poliseno L., Tay Y., Kats L., Pandolfi P. P. (2011). A *ceRNA* Hypothesis: The Rosetta Stone of a Hidden RNA Language?. *Cell*.

[9] Atianand M. K., Hu W., Satpathy A. T. (2016). A long noncoding RNA lincRNA-EPS Acts as a transcriptional brake to restrain inflammation. *Cell*.

[10] Mowel W. K., Kotzin J. J., McCright S. J., Neal V. D., Henao-Mejia J. (2018). Control of immune cell homeostasis and function by lncRNAs. *Trends in Immunology*.

[11] Wan J., Jiang S., Jiang Y. (2019). Data mining and expression analysis of differential lncRNA ADAMTS9-AS1 in prostate cancer. *Frontiers in Genetics*.

[12] Fu D., Zhang B., Yang L., Huang S., Xin W. (2020). Development of an immune-related risk signature for predicting prognosis in lung squamous cell carcinoma. *Frontiers in Genetics*.

[13] Lei K., Yong Z., Ye Z. Q. (2007). CPC: assess the protein-coding potential of transcripts using sequence features and support vector machine. *Nucleic Acids Research*.

[14] Sun L., Luo H., Bu D. (2013). Utilizing sequence intrinsic composition to classify protein-coding and long non-coding transcripts. *Nucleic Acids Research*.

[15] Yu G., Wang L. G., Han Y., He Q. Y. (2012). clusterProfiler: an R package for comparing biological themes among gene clusters. *OMICS*.

[16] Nie X., He M., Wang J. (2020). Circulating miR-4763-3p is a novel potential biomarker candidate for human adult fulminant myocarditis. *Molecular Therapy - Methods & Clinical Development*.

[17] Irizarry R. A., Bolstad B. M., Collin F., Cope L. M., Hobbs B., Speed T. P. (2003). Summaries of Affymetrix GeneChip probe level data. *Nucleic Acids Research*.

[18] Alibés A., Yankilevich P., Cañada A., Díaz-Uriarte R. (2007). IDconverter and IDClight: conversion and annotation of gene and protein IDs. *BMC Bioinformatics*.

[19] Barrett T., Wilhite S. E., Ledoux P. (2013). NCBI GEO: archive for functional genomics data sets--update. *Nucleic Acids Research*.

[20] Ritchie M. E., Phipson B., Wu D. (2015). Limma powers differential expression analyses for RNA-sequencing and microarray studies. *Nucleic Acids Research*.

[21] Aubert J., Bar-Hen A., Daudin J. J., Robin S. (2004). Determination of the differentially expressed genes in microarray experiments using local FDR. *BMC Bioinformatics*.

[22] Babicki S., Arndt D., Marcu A. (2016). Heatmapper: web-enabled heat mapping for all. *Nucleic Acids Research*.

[23] Saijo T., Chen J. H., Chen S. C. (2014). Anti-granulocyte-macrophage colony-stimulating factor autoantibodies are a risk factor for central nervous system infection by Cryptococcus gattii in otherwise immunocompetent patients. *MBio*.

[24] Li G., Zhang H., Wan X. (2014). Long noncoding RNA plays a key role in metastasis and prognosis of hepatocellular carcinoma. *BioMed Research International*.

[25] Eades G., Zhang Y.-S., Li Q.-L., Xia J.-X., Yao Y., Zhou Q. (2014). Long non-coding RNAs in stem cells and cancer. *World Journal of Clinical Oncology*.

[26] Amaral P. P., Mattick J. S. (2008). Noncoding RNA in development. *Mammalian Genome*.

[27] Cesana M., Cacchiarelli D., Legnini I. (2011). A long noncoding RNA controls muscle differentiation by functioning as a competing endogenous RNA. *Cell*.

[28] Cogill S. B., Wang L. (2014). Co-expression network analysis of human lncrnas and cancer genes. *Cancer Informatics*.

[29] Chen R., Jiang T., She Y. (2018). Comprehensive analysis of lncRNAs and mRNAs with associated co-expression and ceRNA networks in C2C12 myoblasts and myotubes. *Gene*.

[30] Aaron P. A., Jamklang M., Uhrig J. P., Gelli A. (2018). The blood-brain barrier internalises Cryptococcus neoformans via the EphA2-tyrosine kinase receptor. *Cellular Microbiology*.

[31] Lee K.-T., Byun H.-J., Jung K.-W., Hong J., Cheong E., Bahn Y.-S. (2014). Distinct and redundant roles of protein tyrosine phosphatases Ptp1 and Ptp2 in governing the differentiation and pathogenicity of Cryptococcus neoformans. *Eukaryotic Cell*.

[32] Tian S., Wan Y., Qin G., Yong X. (2006). Induction of defense responses against Alternaria rot by different elicitors in harvested pear fruit. *Applied Microbiology and Biotechnology*.

[33] Partida-Sanchez S., Desai B. N., Schwab A., Zierler S. (2021). Editorial: TRP channels in inflammation and immunity. *Frontiers in Immunology*.

[34] Wang X., Lin L., Lan B. (2020). IGF2R-initiated proton rechanneling dictates an anti-inflammatory property in macrophages. *Science Advances*.

[35] Yan H., Zheng G., Qu J. (2019). Identification of key candidate genes and pathways in multiple myeloma by integrated bioinformatics analysis. *Journal of Cellular Physiology*.

