# Supplementary material for: Identification of lncRNAs and Their Regulatory Relationships with mRNAs in Response to Cryptococcus neoformans Infection of THP-1 Cells
Source: Biomed Res Int. 2022 Mar 26;2022:5532118. doi: 10.1155/2022/5532118 (PMC8976626; doi:10.1155/2022/5532118)
Supplement: Supplementary Materials — Supplementary 1. Identified lncRNAs and mRNAs that are differentially expressed along with the logFC values and P values. [file 5532118.f1.docx]

Supplementary Table S1. List of primers for quantitative qRT-PCR

|  | F | R |
| --- | --- | --- |
| SMIM34B | ACTTCCAGGTGCTTCGAGAA | AGTCACCTCCTTGTATGCCC |
| IGF2 | AGGACCCCAGAAATCACAGG | CTCTGTCCTCCCCTCCTTTG |
| HIC1 | GTGGGATGGGGTAAGGGAAA | ACAGTGAGAATGGGTGTGGT |
| TRPV3 | TGGTAGAGTTGCTGGTGGAG | GTACTCGGCGTTGATGAACC |
| RHOXF2 | CTCGCAGTGCAGATTTGGTT | AAACACAGGCTGCTGGAATG |
| OR4C6 | GGTGGCTGATGGTGTGATTC | TTTCCACAGCTCCAGGTTCT |
| PATE2 | AGTCTTTCTGCTCTGCCCAT | TGCTCTGCCCTTTCCTTGTA |
| CCL18 | AGGCCAGGAGTTGTGAGTTT | TATAGACGAGGCAGCAGAGC |
| EPCAM | CAGAAGGAGATCACAACGCG | TCCAGATCCAGTTGTTCCCC |
| GCM2 | TATTCCCCACGAGCCAGTTT | TTCACATTTCCCTGCCTCCT |
| MSTRG.22143(ARIH2) | AACAGCACCTGGCAGAAATG | TATTGGCCTGGGCTTAGACA |
| MSTRG.7645(AC048341) | GGAGAGCGATTATGAGCCTTG | CGTTCTGCACTGTGAAACTGGA |
| MSTRG.32644(MFSD14C) | AGGTGCCTTGTGTGTTGGGA | GCAAATGGTGCTTTCGTCTCT |
| MSTRG.14064(VAT1) | TGCTGGACTCACAGAGATTGG | TTTCACCTCTGCCTCATCTTTC |
| MSTRG.13688(NSRP1) | AAGGTAAGGGCCCAACTTCA | GCAACACCAAGACAATTCAAAGG |
| MSTRG.31823(AC067930) | CCTCCACCACACACTGAATCTG | AGGTACTTCCTGGGTGTTCTGAG |
| MSTRG.27113(HIST1H2AC) | AGTAAACTTTCTCCATTTCCCACC | AGTGAATGAATACATCAGGTCCCT |
| MSTRG.6371(RDX) | TTTCAAAGGCTGTATGTAGTGTGG | AACTCTCAGAACAGTGCAAGGC |
| MSTRG.1601(DPYD-AS1) | GCTCTGTGGACTGATATCCCTTT | CCTTCTCTATCCCACATTATCACCA |
| MSTRG.32392 | CATCACCCAAGTCAGACAAACA | CCTATCCACTTCCCTGGCTCT |

F, forward primer; R, reverse primer.
